# Notes and News

**Published:** 1890-11-08

**Authors:** 


### Notes and News.

Collected and Communicated.

**Parcels containing Christmas gifts for the adult patients in hospitals may be addressed "Christmas Parcel, care of The Hospitals' Association, Norfolk House, Norfolk Street, Strand, London." We have to acknowledge with thanks four shirts from "Nellie," and a pair of warm stockings from "H. M." One Sister sends word that she specially needs warm petticoats; another says her patients are always glad of knitted waistcoats or Cardigan jackets. We will gladly receive parcels up to December 17th.**

"The Mayor and Corporation in Church"; well! is that such an extraordinary proceeding that papers should placard it in large letters? What a heathen Corporation, one thought walking through Hull on October 26th, that all this stir should be made about their attending divine service. But after all the occasion was a special one, and it was because it was Hospital Sunday that such a stir was made on the subject. It is just possible that the Mayor goes to church on other Sundays in his private capacity.

In spite of somewhat depressing weather, Liverpool managed to give a very enthusiastic reception to the Duke of Clarence and Avondale on the occasion of the opening of the new Royal Infirmary. There is a saying, "Liverpool loves a lord," but Liverpool simply adores a Prince; and not only was his Royal Highness well received personally, but gold was showered on the institution which brought Royalty to the town. The infirmary is opened free of debt. Brief speeches were made by Colonel Brown, M.P., the Earl of Derby, and others, and the Duke, after inspecting the building, named the women's medical ward the "Clarence" Ward. In the evening there was a ball and a gorgeous supper, in both of which events the Duke of Clarence and Avondale played his part. But, alas! already the decorations are dead, the dancing is almost forgotten, and the supper is a thing of the past. But the infirmary remains; a huge temple of humanity, pleading silently and proudly for the sick and suffering it is sheltering within its walls.

With the fall of the leaf we have got the usual number of epidemics; typhoid and diphtheria are rife, not only in the towns, but also in country districts. The medical wards of most of the London hospitals are full of typhoid cases, and the mere fact that Lady Rosebery and Lady Jersey are both down with the disease, shows its wide prevalence. The outbreak of diphtheria at North Cheam is now happily got in hand. It would never have assumed such a disastrous aspect had there been an isolation hospital available. There are two isolation hospitals within a few miles of the village, but red tape closed their doors. However, two trained nurses were secured, and the disease is no longer spreading. Colds and chills should be especially avoided at this time of the year.

The Bishop of Ripon is always eloquent, and he was at his best in his lecture in aid of the Leeds Workpeople's Hospital Fund. He took for his subject, "The Ministry of Great Races." The Bishop said he wished to draw a conclusion by pointing this moral—that the ministry of the world was but the ministry of great men, though the ministry of great men was not the only ministry of the world. While it was true that God had ministered to the world through its great men, it was also true that the world had been ministered to by masses of mankind, which we called great races. Great men might, as in a battle, have directed those masses, but it had been allotted to the common soldiers to die, and though they lay down their bones in unknown graves, they were not one whit less great, since they also were the benefactors of mankind. It would be a great and glorious heritage if we, though our names should never be known, had done our part in the great work of life.

The Derby and Derbyshire Infirmary celebrated its anniversary with a service at All Saints' Church first, and an annual meeting afterwards. The sermon was preached by the Bishop Suffragan of Derby, and was very practical. He said that during the eighty-one years of the Infirmary's existence it had never passed through a more critical period than it was passing through now. Individuals, as they knew, had had their crises in life, and it was the same with institutions. If the development in sanitary science and laws of health, which, they must never forget, were the laws of God, showed any defect, it was clearly their business as men and Christians to endeavour to remedy it. He proceeded to urge upon them the necessity of supporting the Infirmary to the best of their means, and observed that he trusted the day would never come when hospitals and kindred institutions would be maintained by the State, for it would be a national disgrace.

The Dick Whittington of the future had better go to Sheffield, for gold must be most abundant in a town where committees carelessly forget trifling sums of £6,000 or £7,000. At the quarterly meeting of the Public Hospital and Dispensary at Sheffield, Mr. Lockwood, after reading the minutes of a previous meeting, said that an omission had been made in the last annual report. Among the list of subscribers to the new building fund they omitted to mention the name of Mr. Bernard Wake, who had given over £6,000 for the proposed new out-patients' department. Are you astonished, dear readers? Well, then, just hear this also. Towards the end of the meeting Mr. Lockwood said he had discovered another omission in the annual report. They received notice last year of a magnificent legacy from the late Miss Ray, which could not be shown in the report, as they only notified the receipt of legacies when the money was actually received. This was one of the largest bequests that had ever been made to the institution, amounting, as it did, to between £10,000 and £11,000. Such is the *insouciance* of Sheffield!

THE following vacancies are announced this week, the amount of the salary in each case being given after the name of the institution :—

**Resident Medical Officers.**

Western Dispensary, Westminster, Resident Medical Officer—£100, rooms, &c.
Radcliffe Infirmary, Oxford, House Surgeon—£80, board, &c.
Devon and Exeter Hospital, House Surgeon—£120, board, &c.
Wilts County Asylum, Assistant Medical Officer—£100, board, &c.
Carnarvonshire Infirmary, House Surgeon—£100, board, &c.
Cardiff Infirmary, Assistant House Surgeon—Board, &c.

**Non-Resident Medical Officers.**

Borough of South Shields, Medical Officer of Health—£280.
Ancoats Hospital, Manchester, Honorary Physician.
Belgrave Hospital for Children, Surgeon to Out-patients.
Victoria Hospital for Sick Children, Assistant Surgeon and Assistant Physician.
King's College Hospital, Assistant Physician.
University College, Jodrell Professor of Comparative Anatomy and Zoology.

---

THE *Echo* did us the honour on October 21st of reprinting "Philanthropy in Action," and again on 4th inst. "Hospital Choirs," but it had not the honour to state whence it derived the articles. It is positively amusing on Friday and Saturday evenings to see how paragraph after paragraph in the second-class evening papers is culled from our pages without acknowledgment; it is positively pleasing to see like quotations in the first-class papers—with acknowledgments.

---

IT seems from the plans of the new cottage hospital at Bromsgrove that the buildings will consist of a central block and two side wings connected by corridors. The wings will form the male and female wards respectively; they will be 35ft. by 18ft. 6in. by 12ft. 6in. in height, and they will each have six beds. The central block will have a handsome entrance, with vestibule and entrance hall, on either side of which will be a day room and the matron's room. A passage will lead from the central hall to the rear, and opening upon it will be linen and splint stores, dispensary, operating room, surgical ward, kitchen, staircase, &c. The buildings will be one storey in height, except the front portion of the central block, which will be carried up two storeys, and will provide a large bed-room, which will also be available as an additional ward if required, three bed-rooms for the matron and the nurses, and a store-room. The architect is Mr. John Cotton, of Birmingham.

---

A SCHEME for the remedy of hospital abuse has been brought before the Birmingham Committee by Mr. Griffith, of the Charity Organisation Society. This is the scheme in brief: "Divide the town into districts—for example the seven Parliamentary districts—establish a branch office of the Charity Organisation Society in each district, appoint a local committee of earnest, sympathetic, and prudent men, and, perhaps, ladies, supported by additional subscribers and increased subscriptions to the parent society; establish in each district, on the most approved and well-tried principles, a provident dispensary, managed by a committee, including representatives of the members, all of which should, in a very short time, as in Manchester, be self-supporting. Open your hospitals free to the class for which they are intended; abolish all subscribers' notes and registration fees, and afford every facility for the quick and efficient treatment of the applicants, without wasting their time and that of the doctors; determine some test of eligibility, either a wage test, as in Manchester, or some other equally good; institute a system of inquiry and investigation into the circumstances of each applicant, to be conducted by the local committee of the Charity Organisation Society." This is the scheme in briefer: "Put yourself entirely into the hands of the C.O.S."

